# Alteration of Gut Microbiota: New Strategy for Treating Autism Spectrum Disorder

**DOI:** 10.3389/fcell.2022.792490

**Published:** 2022-03-03

**Authors:** Jiayin Liu, Zhanyuan Gao, Chuanqi Liu, Tianyao Liu, Junwei Gao, Yun Cai, Xiaotang Fan

**Affiliations:** ^1^ Department of Military Cognitive Psychology, School of Psychology, Third Military Medical University (Army Medical University), Chongqing, China; ^2^ Battalion 5th of Cadet Brigade, Third Military Medical University (Army Medical University), Army Medical University, Chongqing, China

**Keywords:** gut microbiota, autism, therapeutic interventions, microbiota–gut–brain, core symptoms

## Abstract

Autism spectrum disorder (ASD) is defined as a complex heterogeneous disorder and characterized by stereotyped behavior and deficits in communication and social interactions. The emerging microbial knowledge has pointed to a potential link between gut microbiota dysbiosis and ASD. Evidence from animal and human studies showed that shifts in composition and activity of the gut microbiota may causally contribute to the etiopathogenesis of core symptoms in the ASD individuals with gastrointestinal tract disturbances and act on microbiota-gut-brain. In this review, we summarized the characterized gut bacterial composition of ASD and the involvement of gut microbiota and their metabolites in the onset and progression of ASD; the possible underlying mechanisms are also highlighted. Given this correlation, we also provide an overview of the microbial-based therapeutic interventions such as probiotics, antibiotics, fecal microbiota transplantation therapy, and dietary interventions and address their potential benefits on behavioral symptoms of ASD. The precise contribution of altering gut microbiome to treating core symptoms in the ASD needs to be further clarified. It seemed to open up promising avenues to develop microbial-based therapies in ASD.

## 1 Introduction

Autism spectrum disorder (ASD) is defined as a complex heterogeneous disorder with pervasive neurodevelopmental disability (Arakawa, 2020; Valentino et al., 2021). The clinical characteristics of ASD include impairments in social interaction and communication, as well as restricted interests and repetitive behaviors (Supekar et al., 2021). ASD generally appears early in childhood and is diagnosed before the age of three in most cases. The incidence of ASD has dramatically increased in recent years, with an estimated prevalence of 1%–2% according to numerous studies conducted in Asia, Europe, and North America (Elsabbagh et al., 2012; Valentino et al., 2021). Gender ratios have been reported to have a higher prevalence in males than in females, ranging from 2:1 to 5:1 (Brunissen et al., 2021). It is obvious that ASD intervention has become a very urgent need for public health to solve the cost and complexity of medical care. Social skill interventions based on behavioral therapy and pharmacological interventions aimed at comorbidities have been adopted in patients with Asperger’s syndrome and high-functioning autism (Corbett et al., 2019; Romagnoli et al., 2019; Frolli et al., 2020; Oshima et al., 2020). However, due to the complexity of the pathogenesis mechanism underlying the disease and limited animal models, there is a lack of Food and Drug Administration (FDA)-approved drug to treat the core symptoms of ASD effectively (Donegan and Lodge, 2020).

Currently, many disparate mechanisms, such as decline in neurogenesis, an imbalance in excitatory/inhibitory neurotransmission, and dysregulated immune response, have been hypothesized to be involved in the onset and development of ASD-related behavior (Selimbeyoglu et al., 2017; Cai et al., 2018; Cristiano et al., 2018; Cai et al., 2019; Zhang et al., 2019; Dong et al., 2020; Poornimai Abirami et al., 2020; Sacai et al., 2020; Adhya et al., 2021; Zhong et al., 2020; Baranova et al., 2021; Cast et al., 2021). Notably, the emerging microbial knowledge has pointed that the complex intestinal microbial community and their metabolic consequences may contribute to ASD etiopathogenesis of core symptoms, such as in central nervous system (CNS)-driven behaviors, in a very powerful way (Oh and Cheon, 2020). The communication between the brain and the gut is commonly known as the microbiota–gut–brain axis, which is suggested to be involved in maintaining physiological homeostasis (Oh and Cheon, 2020). The bi-directional communication between the gut and brain occurs *via* neuroimmune and neuroendocrine signaling, the vagus nerve, and gut microbial metabolites (Ersöz Alan and Gülerman, 2019). It is increasingly understood that gut microbiota contributes to the regulation of social behavior *via* the microbiome–gut–brain axis (Dinan and Cryan, 2017). More recently, several lines of studies suggest that microbial-based therapeutic interventions such as fecal microbiota transplantation (FMT), antibiotics, and probiotics have emerged as novel therapeutic strategies to improve core and associated ASD symptoms (Johnson et al., 2020; Martínez-González and Andreo- Martínez, 2020; Davies et al., 2021).

This review article will describe the involvement of gut microbiota and their metabolites in the ASD pathogenesis and progression. Existing literature relevant to therapeutic interventions based on alteration of gut microbiota in ASD appears to fall within the following four domains: prebiotic/probiotic/synbiotic, FMT, dietary interventions, and antibiotics. The literature on each domain will be reviewed and microbial-based therapeutic interventions in ASD patients will be highlighted in a comprehensive way.

## 2 Microbiota–Gut–Brain Axis in ASD

The human gut is an integral part of human physiology and metabolism and harbors trillions of microbes including bacteria, eukaryotic microorganisms, viruses, and archaea, commonly referred to as the gut microbiota (Moszak et al., 2020; Ogunrinola et al., 2020). The distinctive microbial signature increases exponentially from the proximal to the distal portion of the gastrointestinal tract (GIT) and varies according to many factors such as age, genetics, and nutrition (Dhar and Mohanty, 2020; Giles and Couper, 2020; Sakkas et al., 2020; Seitz et al., 2020). Moreover, short-chain fatty acids (SCFAs), the microbial metabolites produced by gut bacteria, have been indicated to exert profound effects on the gut, brain, and behavior and contribute to ASD pathology (Tran and Mohajeri, 2021). Notably, SCFAs mainly contain butyrate, propionate, and acetate; possess neuroactive properties; and can operate on immunomodulatory system (Parada Venegas et al., 2019). Gut microbiota, including the four major phyla (*Bacteroidetes*, *Firmicutes*, *Proteobacteria*, and *Actinobacteria*) and two minor phyla (*Verrucomicrobia* and *Fusobacteria*), is increasingly recognized as a metabolic organ, contributing to the maintenance of individual homeostasis through nutrition, immune regulation, and systemic inflammation pathways (Guo et al., 2018; Adak and Khan, 2019; Khan et al., 2020).

An increasing amount of evidence has shown that GIT symptoms, ranging from constipation to diarrhea, are more frequently reported in autistic children than in typically developing (TD) children (Lefter et al., 2019; Sanctuary et al., 2019; Żebrowska et al., 2021). Moreover, the presence of GI symptoms correlates with the apparent behavioral manifestations in ASD children such as anxiety, self-injury, and aggression, indicating that exacerbated autism symptoms are explained partially by the underlying GI problems (Neuhaus et al., 2018). There is growing scientific evidence that GI comorbidity may have secondary effects on problematic behaviors of ASD individuals (Lasheras et al., 2020). Meanwhile, significant alterations in the stability, diversity, composition of the gut microbiota, and several microbial metabolic pathways were apparently detected in ASD children (Ho et al., 2020). Disrupted intestinal permeability and evidence of a systemic and intestinal inflammation were also detected in ASD subjects (Riccio and Rossano, 2019; Yang et al., 2020).

It is widely accepted that antibiotics affect the composition and diversity of the gut microbiota (Angelucci et al., 2019). This is supported by the evidence that early exposure to antibiotics may result in long-term disturbances in the gut microbiota of ASD children, involved in the rising recurring GI symptoms and changes in the microbiota composition (Vargason et al., 2019). More recently, it was revealed that transplantation of the fecal sample extract from ASD children into pregnant rats caused typical behavioral characteristics of ASD in offspring, recapitulating the abnormal gut microbiota of ASD (Qi et al., 2021). Altogether, these data from animal and clinical studies suggested that abnormal gut microbiota could contribute to core and associated ASD symptoms.

Gut dysbiosis refers to shifts in composition and activity of the gut microbiota, leading to detrimental effects on host health (Shanahan et al., 2021). Gut dysbiosis could be induced by genetics, environmental factors, diet, disease, stress, and age, which might destroy the intestinal mucosal barrier and cause amyloid and lipopolysaccharide (LPS) leak, then further increase microbial molecules in blood and activate the hypothalamic–pituitary–adrenal axis; thereby triggering a systemic and CNS inflammation with disruption of the blood–brain barrier (Cristofori et al., 2021). An increasing amount of evidence has established a close relationship between gut microbiota dysbiosis and GI symptoms in ASD children (Wang et al., 2011; Gorrindo et al., 2012). Intriguingly, gut dysbiosis also directly lead to ASD-like behavior (Vuong and Hsiao, 2017). Thinking along these lines, in ASD individuals, microbiota dysbiosis promotes toxins and bacterial products to enter into the bloodstream *via* damaged intestinal barrier. The brain, in turn, modulates gut peristalsis and sensory and secretion functions, linking sympathetic glutamatergic neurons in the CNS to the gut through the vagus nerve (Zhu et al., 2018). Thus, inflammation and local damage in the brain also leads to gut dysbiosis, which become a promising therapeutic target. Likewise, we have recently reported that traumatic brain injury in mice induced significant gut dysbiosis such as increasing enrichment of the class Alphaproteobacteria and the families Porphyromonadaceae and Pseudomonadaceae, which are associated with neuroinflammation, brain edema, neuronal loss, and sensorimotor deficits (Ma et al., 2019). *Lactobacillus acidophilus* treatment resulted in the remodeling of the gut microbiota and rescuing CNS dysfunction (Ma et al., 2019).

## 3 Gut Dysbiosis in ASD Animal Models and Patients

### 3.1 Gut Dysbiosis in ASD Animal Models

Animal studies have also indicated that gut microbiota alterations lead to profound changes in ASD-like behaviors. The composition of gut microbiota in rodents showing features of ASD was detected in models of environmental risk factors such as valproic acid (VPA) exposure, maternal immune activation (MIA), maternal high-fat diet (MHFD) and p-Cresol exposure, idiopathic model for autism (BTBR mice), and monogenetic mutation mouse models of autism such as *Shank3* KO mice*,* NLGN3^R451C^ mutants, *and EphB6* KO mice (Hsiao et al., 2013; Buffington et al., 2016; Newell et al., 2016; Liu et al., 2018; Tabouy et al., 2018; Cai et al., 2019; Hosie et al., 2019; Li et al., 2020; Bermudez-Martin et al., 2021; Zhong et al., 2020). The altered gut microbiota composition in ASD animal models is summarized in Table 1.

**TABLE 1:** Altered gut microbiota composition in ASD animal models (↑ = increased, ↓ = decreased).

Animal models	Findings	References
Gut dysbiosis in ASD animal models induced by environmental risk factors		
VPA-exposed Sprague–Dawley rats	*α-Proteobacteria*↑, *Eubateriaceae*↑, Enterobacteriaceae↓, *Rikenellaceae*↑, *Staphylococcaceae*↑, *Anaerotruncus*↓, *Anaerofustis*↑, *Proteus*↑, *Staphylococcus*↑, *Allobaculum*↑, Males:*Bacteroidetes*↑, *Bacteroidia*↑, *α-Proteobacteria*↑, Females: *Actinobacteria*↑, *Allobaculum* ↑, *Odoribacter*↑, *Staphylococcus*↑, *Bifidobacterium*↑, and *Candidatus Arthromitus*↑	Liu et al. (2018)
VPA-exposed male Wistar rats	Fourth week: *Prevotellaceae*↑, *Streptococcaceae*↑, *Desulfovibrionaceae*↑, *Ruminiclostridium 6*↓, *Lactobacillus*↓, *Ruminococcaceae UCG-010*↓, *Ruminococcaceae UCG-004*↓, and *Candidatus Arthromitus*↓, Eighth week: *Marvinbryantia*↓ and *Helicobacter*↓	Kong et al. (2021a)
VPA-exposed male and female BALB/C mice	*Bacteroidales*↓, *Deltaproteobacteria↓*, *Clostridiales*↑, and *Erysipelotrichales*↑, Males*: Alistipes*↑, Enterorhabdus↑, Erysipelotrichalis↑, *Lactobacillales*↑, and *Mollicutes*↑	de Theije et al. (2014)
MIA mouse model	Porphyromonadaceae↑, Erysipelotrichaceae↓, Alcaligenaceae↓, Prevotellaceae↑, Ruminococcaceae↓, *unclassified Bacteriodales*↑, and Lachnospiriceae↑	Hsiao et al. (2013)
MHFD mouse model	*Lactobacillus reuteri*↓, *Bifidobacterium pseudolongum*↓, *Parabacteroides distasonis*↓, *Bacteroides uniformis*↓, *Olsenella unclassified*↓, *Collinsella unclassified*↓, *Lactobacillus johnsonii*↓, and *Helicobacter hepaticus*↓	Buffington et al. (2016)
p-Cresol-exposed mice	*Duncaniella dubosii*↑, *Barnesiella* sp.↑, *Anaerobium* sp.↑, *Muribaculaceae bacterium*↑, *Turicimonas muris*↑, *Eisenbergiella* sp.↓, *Lacrimispora saccharolytica*↓, *Anaerobium* sp.↓, *Clostridiaceae* bacterium↓, and *Ruthenibacterium lactatiformans*↓	Bermudez-Martin et al. (2021)
Gut dysbiosis in BTBR mouse model of idiopathic autism		
BTBR mice	*Clostridium* cluster XI↑, *Bacteroidetes*↑, and *Firmicutes*↓	Newell et al. (2016)
BTBR mice (12 months of age)	*Dehalobacterium*↓, *Bacteroide*s↑, *Parabacteroides*↑, Females: *Prevotella*↑, *Coprobacillus*↑, *Sutterella*↑, *Akkermansia* (*muciniphila*)↑, *Oscillospira* ↓, *Males*: *Lactobacillus*↑, *Ruminococcus*↓, unclassified member of Helicobacteriaceae↑, and *Desulfovibrio*↓	Coretti et al. (2017)
Adult BTBR male mice	*Akkermansia*↑, *Bacteroides*↑, *Bilophila*↑, *Desulfovibrio*↓, *Bifidobacterium*↓, Rikenella↓, Blautia↓, Odoribacter↓, and Parabacteroides↓	Golubeva et al. (2017)
Gut dysbiosis in monogenetic mutation mouse models of autism		
*Shank3* KO mice	*Lactobacillales*↓, *Lactobacillaceae*↓, *Veillonellaceae*↑, *Bacteroides*↓, *Lactobacillus*↓, *Bacilli*↓, *Veillonella*↑, Coprococcus↓, *Prevotella*↓, *Acetobacter*↓, and Turicibacter↓	Tabouy et al. (2018)
NL3^R451C^ mice	*Firmicutes*↑, *Clostridia*↑, and *Candidate*↓	Hosie et al. (2019)
*Fmr1* KO2 mice	*Allobaculum*↑, *Akkermansia*↑, *Sutterella*↑, *Odoribacter*↑, *Desulfovibrio*↑, *Turicibacter*↑, *Bifidobacterium*↑, *Flexispira*↓, *Bacteroides*↓, and *Oscillospira*↓	Altimiras et al. (2021)
15q dup mice	Species diversity of the microbiome↓; number of OTUs↓	Septyaningtrias et al. (2020)
*EphB6* KO mice	*Deferribacteres*↓ and *Mucispirillum*↓	Li et al. (2020)

### 3.2 Gut Dysbiosis in ASD Animal Models Induced by Environmental Risk Factors

Mice exposed to VPA *in utero* exhibited altered gut microbiota composition and presented with a decrease of *Bacteroid*s, *Deltaproteobacteris*, and *Erysipelotrichales* as well as an increase of *Clostridiales* (de Theije et al., 2014). An impact of gender on the gut microbiota composition of VPA in utero-exposed mouse offspring was observed, characterized by increased levels of *Alistipes*, *Enterorhabdus*, *Mollicutes*, *Lactobacillales*, and *Erysipelotrichalis* in males (de Theije et al., 2014). Liu et al. (2018) have confirmed that sex-specific differences in gut microbiota composition also existed in VPA exposed rats. VPA-exposed rats displayed decreased microbial diversity and increased in the abundance of *α*-Proteobacteria, Eubateriaceae, Rikenellaceand, and Staphylococcaceae. At the genus level, VPA exposure increased the abundance of the genera *Allobaculum*, *Anaerofustis*, *Proteus*, and *Staphylococcus* significantly (Liu et al., 2018). One recent study has further reported that there was a significant difference in the composition of gut microbiota and SCFA levels between autistic-like and healthy rats during weaning and sexual maturation (Kong et al., 2021a). Evidence has indicated that the MIA offspring display damaged GI integrity, microbiota dysbiosis, and changes in serum metabolites that are similar to ASD endophenotypes (Hsiao et al., 2013). Principal coordinate analysis (PCoA) showed robust differences in the diversity of *Clostridia* and *Bacteroidia* between MIA offspring and controls. MIA offspring displayed increased abundance in the families of *Lachnospiraceae*, *Porphyromonadaceae*, and *Prevotellaceae*, although the species richness was not altered. Moreover, specific Lachnospiraceae, along with other *Bacteroidial* species, might be involved in the pathogenesis of MIA-induced autistic behavior and impact MIA-associated GI abnormalities (Hsiao et al., 2013). In the MHFD mouse model for autism, the diversity of microbiota was lower than that in the control mice, with significant reductions in *Lactobacillus*, *Parabacteroides*, *Helicobacter*, and *B. Uniformis* (Buffington et al*.,* 2016). Mice exposed to the microbial metabolite p-Cresol for 4 weeks in drinking water exhibited social interaction deficits and stereotypies, reminiscent of ASD core symptoms in humans (Bermudez-Martin et al., 2021). Moreover, the microbiota from p-Cresol-treated mice induced social behavior deficits and significant divergences in microbial composition when transplanted to untreated recipients (Bermudez-Martin et al., 2021). Importantly, a growing body of independently replicated findings revealed that germ-free (GF) mice, devoid all microorganisms in or on their bodies, displayed impaired social behavior, which indicated that the absence of bacterial colonization during development exerts a detrimental effect on animal social behavior (Stilling et al., 2015; Stilling et al., 2018). Additionally, it has been further supported by a study that the GF mice colonized with microbiota from children with ASD promoted ASD-relevant behaviors in GF mice and affected the distribution and composition of intestinal microorganism (Xiao et al., 2021).

### 3.3 Gut Dysbiosis in BTBR Mouse Model of Idiopathic Autism

Noteworthy, BTBR mice displayed a global alteration of microbial communities of cecal and fecal samples, suggesting that this model may be useful to interpret gut–brain interactions in ASD (Newell et al., 2016; Coretti et al., 2017; Golubeva et al., 2017). Measurement of 16S rRNA sequences showed BTBR mice harbored a different fecal microbial community in gut such as increased *Clostridium* cluster XI, *Bacteroidetes*, and decreased *Firmicutes* (Newell et al., 2016). Moreover, BTBR mice fed a chow diet showed a significantly increased level of *A. muciniphila*, the predominant representative of the *Verrucomicrobia* phylum within the GI tract in both cecal and fecal matter, which are linked to maintaining homeostasis of mucus secretions (Newell et al., 2016). In particular, *Bacteroides*, *Parabacteroides*, *Sutterella*, *Dehalobacterium*, and *Oscillospira* genera as key drivers of sex-specific gut microbiota composition have been identified in BTBR mice (Coretti et al., 2017). The genera *Bacteroides* and *Oscillospira* were indicated to be related to pathological traits, which might help to understand the sex-induced alteration of behavior, gut integrity, and colon immunological state in BTBR mice (Coretti et al., 2017). Similarly, Golubeva et al. (2017) showed a substantially reduced gut bacterial diversity in the BTBR cecum. PCoA revealed that BTBR mice displayed an increase in *Bacteroidetes* and a decrease in *Firmicutes* at the phylum level; an increase in *Akkermansia*, *Bacteroides*, and *Bilophila* genera; and a reduction in *Bifidobacterium* and *Desulfovibrio* (Golubeva et al., 2017).

### 3.4 Gut Dysbiosis in Monogenetic Mutation Mouse Models of Autism

In the case of autism, studies have shown autism-associated genes also influence the microbiome (Tabouy et al., 2018; Hosie et al., 2019; Liu et al., 2021). Using the LefSe bioinformatic tool, Tabouy et al. (2018) found that the bacterial richness was decreased in the *Shank3* KO mice, with a decrease in the relative abundance of members of the class *Bacilli*, order *Lactobacillales*, family *Lactobacillaceae*, and genus *Lactobacillus*. At the genus level, *Coprococcus*, *Bacteroides*, *Acetobacter*, *Turicibacter*, and *Prevotella* were also decreased in the *Shank3* KO mice, while the family *Veillonellaceae* and genus *Veillonella* were increased in the *Shank3* KO mice (Tabouy et al., 2018). Neuroligin 3 (NLGN3) has been confirmed as risk alleles for ASD (Jamain et al., 2003). Interestingly, *NLGN3* KO mice displayed gut dysfunction such as decreased colonic smooth muscle tone and altered colonic motility. Furthermore, NLGN3^R451C^ mutants showed altered fecal microbial communities including increased three operational taxonomic units (OTUs) belonging to the phylum *Firmicutes*, class *Clostridia*, as well as one decreased OTU from the *Candidate* phyla (Hosie et al., 2019). Fragile X syndrome (FXS) is considered as the leading monogenetic cause of autism, and the FXS phenotype could be well recapitulated in *Fmr1* KO2 mice. Using 16S ribosomal RNA gene sequencing, Altimiras et al. (2021) identified bacterial species alterations in the gut microbiome of *Fmr1* KO2 mice including an increase in the *Firmicutes*, *Bacteroides*, and *Verrucomicrobia* phyla and *Sutterella* and *Akkermansia* genera and a decrease in the *Prevotella* genus. Septyaningtrias et al. (2020) also found a significant reduction in species diversity of the microbiome and OTU number in fecal samples in a mouse model for ASD with the human 15q11-13 duplication (*15q dup*). *EphB6*, located on chromosome 7q, is identified as a candidate ASD-associated gene. One report has indicated that *EphB6* KO mice displayed altered gut microbial composition in the fecal microbiota including a decreased abundance of the phylum *Deferribacteres*, especially *Mucispirillum* at the genus level (Li et al., 2020).

### 3.5 Gut Dysbiosis in ASD Patients

It has been noted that the gut microbiota stabilization occurs around the age of 2 to 3, a critical period for the ASD onset (Rautava et al., 2012). GI system disorder is a common comorbidity in children with ASD. Disruptions in the gut bacteria profiles may tend to strongly correlate with the increased risk and severity of autism (Zhang et al., 2018). It is supposed that a neurotoxin produced by bacteria reaches the brain *via* the vagus nerve and may lead to communication and social impairments. The altered gut microbiota composition in ASD patients is summarized in Table 2.

**TABLE 2:** Altered gut microbiota composition in ASD patients (↑ = increased, ↓ = decreased).

Study subjects	Findings	References
13 autistic children and 8 control children	*Clostridium↑* and *Ruminococcus* spp.↑	Finegold et al. (2002)
15 autistic children and 8 control children	*C. bolteae↑*, *Clostridium* clusters I↑, and *Clostridium* clusters XI*↑*	Song et al. (2004)
58 autistic children, 12 healthy siblings, and 10 control children	*Clostridium* clusters I↑ and *Clostridium* clusters II↑	Parracho et al. (2005)
33 autistic subjects,7 sibling controls, and 8 non-sibling control subjects	*Bacteroidetes↑*, *Proteobacteria↑*, *Alkaliflexus↑*, *Desulfovibrio↑*, *Acetanaerobacterium↑*, *Bacteroides↑*, *Parabacteroides↑*, *Desulfovibrio* spp*. ↑*, *Bacteroides vulgatus↑*, *Actinobacteira*↓, *Turicibacter Clostridium*↓, *Firmicutes*↓, *Weissella*↓, *Helcococcus*↓, *Alkaliphilus*↓ *Anaerofilum*↓, *Pseudoramibacter*↓, *Ruminococcus*↓, *Streptococcus*↓, *Anaerovorax*↓, *Dialister*↓, *Lactococcus*↓, *Leuconostoc*↓, and *Ethanoligenens*↓	Finegold et al. (2010)
15AUT-GI and 7 control-GI children	*Betaproteobacteria↑*, *Bacteroidetes*↓, and the ratio of *Firmicutes/Bacteroidetes↑*	Williams et al. (2011)
10 autistic children, 9 siblings, and 10 healthy children	*Lactobacillus* spp.*↑*, *Desulfovibrio* spp.*↑*, and *Bacteroidetes/Firmicutes*↓	Tomova et al. (2015)
35 children with ASD and 6 TD children	*Sutterella↑*, *Odoribacter↑*, *Butyricimonas↑*, *Veillonella*↓, *Streptococcus*↓, and *Bacteroidetes/Firmicutes↑*	Zhang et al. (2018)
48 children with ASD and 48 healthy children	*Firmicutes*↓, *Proteobacteria*↓, *Verrucomicrobia*↓, *Bacteroidetes/Firmicutes*↑, *Dialister*↓, *Prevotella*↑, *Bacteroides*↑, *Megamonas*↑, *Escherichia/Shigella*↓, *Lachnospiracea_incertae_sedis*↑, *Clostridium XlVa*↓, *Eisenbergiella*↓, *Clostridium IV*↓, *Flavonifractor*↓*, Haemophilus*↓, and *Akkermansia*↓	Zou et al. (2020)
77 children with ASD and 50 age-matched healthy children	*Unidentified Lachnospiraceae*↑, *Clostridiales*↑, *Dorea*↑, *Erysipelotrichaceae*↑, *Collinsella*↑, *Lachnoclostridium*↑, *Bacteroides*↓, *Faecalibacterium*↓, *Parasutterella*↓, and *Paraprevotella*↓	Ding et al. (2020)
9 autistic children, and 6 healthy children	*Bacteroidales* ↓, *Selenomonadales*↓, *Prevotellaceae*↓, and *Ruminococcaceae*↑	Sun et al. (2019)
143 ASD children, 143 age- and sex-matched TD individuals	*Firmicutes*↑, *Firmicutes*/*Bacteroidetes*↑, *Megamonas*↓, *Proteobacteria*↑, *Actinobacteria*↑, *Bacteroidetes*↓, *Dialister*↑, *Escherichia-Shigella*↑, *Bifidobacterium*↑, *Prevotella* 9↓, and *Ruminococcus* 2↓	Dan et al. (2020)
72 ASD and 74 TD children	*Clostridium*↑, *Dialister*↑, *Coprobacillus*↑, and *Faecalibacterium*↓	Wan et al. (2021)
11 ASD and 14 healthy control children	*Actinobacteria*↓, *Bacteroidetes*↑, *Proteobacteria*↑, *Bacteroidetes/Firmicutes*↑, *Actinomycetaceae*↓, *Coriobacteriaceae*↓, *Oscillospira*↑, *Gemellaceae* ↓, *Streptococcaceae*↓, *Faecalibacterium prausnitzii*↑, and *Bifidobacteriaceae*↓	Coretti et al. (2018)

The finding of a microbial element involved in autism pathogenesis was reported in 1998 (Bolte, 1998). Several pieces of evidence have shown that the compositional changes in the gut microbiota are related to alterations in the normal function of the nervous system and behavioral deficits in ASD (Williams et al., 2011; Ding et al., 2020; Ho et al., 2020). Finegold et al. (2002) found differences in *Clostridial* species between children with late-onset autism and control children. Typically, non-spore-forming anaerobes and microaerophilic bacteria were in the intestinal flora of ASD children but absent in control children (Finegold et al., 2002). Consistent with this finding, Song et al. (2004) revealed that *Clostridial* groups such as *Clostridium bolteae* and clusters I and XI in feces significantly elevated in ASD children. Parracho et al. (2005) found the *Clostridium histolyticum* group (*Clostridium* clusters I and II) abundant in the fecal flora of ASD children.

The investigation of the dominant intestinal bacterial phyla indicates that the dysbiosis exhibits a similar trend, primarily characterized by the alterations in the ratio and the composition of the primary bacterial phyla (*Firmicutes*, *Bacteroidetes*, *Fusobacteria*, and *Verrucomicrobia*) (De Angelis et al., 2015). Finegold et al. (2010) found that the phylum level of *Bacteroidetes* and *Proteobacteria*; the genus level of *Alkaliflexus*, *Desulfovibrio*, *Acetanaerobacterium*, *Parabacteroides*, and *Bacteroides*; and the species of *Desulfovibrio* spp. and *Bacteroides vulgatus* were higher in the ASD group than those in controls. Also, from the phylum level, *Actinobacteira* and *Firmicutes* and from the genus level *Weissella*, *Turicibacter*, *Clostridium*, *Anaerofilum*, *Pseudoramibacter*, *Ruminococcus*, *Streptococcus*, *Anaerovorax*, *Dialister*, *Lactococcus*, *Leuconostoc*, *Ethanoligenens*, *Helcococcus*, and *Alkaliphilus* were higher in the control group than those in the ASD group (Finegold et al., 2010). One study in a cohort of young ASD children (2–4 years of age) and age-matched neurotypical healthy controls has indicated that ASD children displayed a significant increase in *Bacteroidetes* and *Proteobacteria* and a decrease in *Actinobacteria* (Coretti et al., 2018). Of note, *Bifidobacterium longum*, the dominant bacterium in infant gut microbiota, was significantly depleted, while *Faecalibacterium prausnitzii*, a late colonizer of healthy human gut and major butyrate producer, was increased in ASD patients (Coretti et al., 2018). Likewise, one recent study reported that the genus level of *Bacteroides*, *Prevotella*, *Lachnospiracea_incertae_sedis*, and *Megamonas* as well as *Bacteroidetes/Firmicutes* increased in autistic children relative to the control children (Zou et al., 2020).

Differently, another study in ASD child cohort has confirmed that gut dysbiosis is characterized by a decrease in Bacteroidetes and ratio of Bacteroidetes to *Firmicutes* and a greater preponderance of Betaproteobacteria in the intestinal biopsy samples (Williams et al., 2011). Tomova et al. (2015) have found a significantly decreased *Bacteroidetes*/*Firmicutes* ratio, but increased counts of *Lactobacillus* spp., detected in ASD children in Slovakia. They also noticed that a trend of increase in *Delsulfovibrio* spp. was especially strongly associated with the severity of autism. Sun et al. (2019) have found a large difference in the abundance of microbiota at the level of family, genus, and species between the ASD group and the healthy control group. Ding et al. (2020) have shown that gut microbiota in ASD children displayed higher biomass, richness, and biodiversity and an altered microbial community structure at the genus level in the ASD group. Notably, three altered intestinal microbiome strains in ASD children, namely, *Erysipelotrichaceae*, *Faecalibacterium*, and *Lachnospiraceae*, were related to the production of butyric acid in the gut and were positively correlated with ASD severity (Ding et al., 2020).

As stated, numerous studies have demonstrated the variation in the gut microbiota of autistic children (Dan et al., 2020; Wan et al., 2021), but there is little consensus on the uniform microbial profile of individuals with ASD and disparities in currently available data. Heterogeneity in population studied, sample collection, and study design all result in variability in microbiome studies. This may be caused by methodological dissimilarities and the differences between the studied objects. Moreover, age is a significant factor leading to this deviation. Patients at different ages have some differences in gut microbiota. In younger children, gut microbiota remains unstable (Rautava et al., 2012). Thus, ongoing research is further needed to identify the association between microbiota alterations and ASD.

### 3.6 Pathogenesis Mechanisms Underlying Gut Dysbiosis Involved in the ASD

**FIGURE 1:**
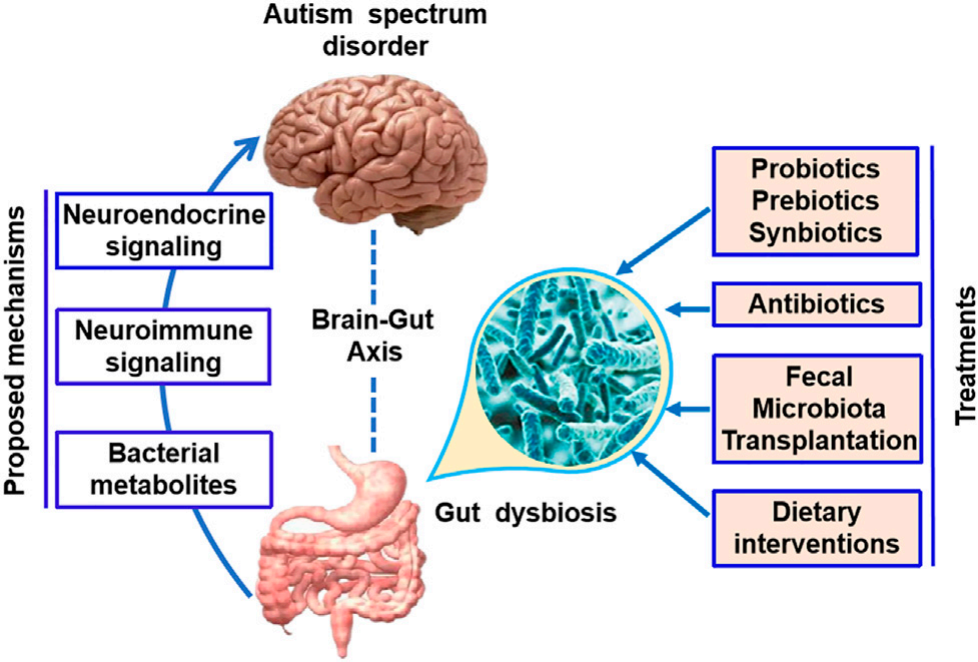
Microbial-based therapeutic interventions in ASD. The gut microbiota has been found to affect brain function through the neuroendocrine signaling, neuroimmune signaling, and bacterial metabolites. Potential microbial-based therapeutic interventions in ASD include prebiotic/probiotic/synbiotic, antibiotics, fecal microbiota transplantation, and dietary interventions.

A variety of studies have indicated that the gut microbiota affects brain function through neuroendocrine signaling, neuroimmune signaling, and bacterial metabolites (Figure 1) (Li et al., 2017).

### 3.7 Neuroendocrine Signaling

The enteric nervous system (ENS) consisting of millions of neurons regulating GI functions is referred to as a “second brain” (Zhang et al., 2014). The ENS may interplay with the intestinal bacteria directly or indirectly. The intestinal microbes affect the biologically active peptides and neuroactive molecules in the gut and blood. Besides, intestinal bacteria play critical roles in the production of neurotransmitters in the intestinal lumen through host biosynthetic pathways, which might be the molecular basis for the alterations in neurotransmitter metabolism (O'Donnell et al., 2020). It has been confirmed that *Lactobacillus* spp. is capable of producing *γ*-aminobutyric acid (GABA) and acetylcholine. Similarly, *Bacillus* spp. and *Serratia* spp. have been found to produce dopamine, while *Candida* spp., *Streptococcus* spp., *Escherichia* spp., and *Enterococcus* spp. produce serotonin (5-hydroxytryptamine, 5-HT), a particularly important brain neurotransmitter, deeply affecting intestinal physiology and the behavior. In support of this, evidence suggests that the microbiota can affect dopamine circuits and vagal sensory neurons in the gut, which is essential for central dopamine function (Travagli et al., 2020). Moreover, Reigstad et al. (2015) also found that the gut microbiota acts through SCFAs, which have the ability to promote the enteric 5-HT production and homeostasis. These produced neurotransmitters can transduce signals to the CNS, then modulate the behavior *via* acting on different neural circuits. The increase in *Clostridales*, along with a decrease in *Dorea*, *Blautia*, and *Sutterella*, could also alter host processing of tryptophan, the precursor of serotonin, and an overabundance of serotonin (Luna et al., 2017). This in turn apparently caused an increase in the intestinal serotonergic effects and serotonin deficiency in the host, which may be associated with deficits in mood and cognitive function seen in ASD (Luna et al., 2017).

### 3.8 Neuroimmune Signaling

It has been suggested that the gut can also interact with the brain through immunological pathways, and the altered host immune responses arising from gut microbiota are closely related to ASD-relevant symptoms (Ashwood et al., 2006; Cristiano et al., 2018). The bacterial cell wall constituents persistently stimulate the innate immune system to produce inflammatory cytokines, consequently keeping a basal state of immune activation at the intestinal mucosal surface and influencing the whole body (Duerkop et al., 2009). Immune responses to toxins generated by pathogenic microbiota and focal inflammation induce an increase in gut permeability. The defects in the gut barrier further cause the toxins and bacterial products to enter into the bloodstream, which might result in brain dysfunction (Yoo et al., 2020).

Luna et al. (2017) found that an increase in *Clostridales*, combined with a decrease in *Dorea*, *Blautia*, and *Sutterella*, could change pro-inflammatory cytokines, which can reach the brain through blood flow. *Bacteroides fragilis* and several members of the genus *Clostridia* mediate an anti-inflammatory action through promoting the production of anti-inflammatory cytokines, such as IL-10 and IL-13, whereas some pathogenic bacteria (*Salmonella typhimurium* and *C. difficile*) trigger the production of inflammatory cytokines (Lombardi et al., 2018). Hydrogen sulfide, which is generated by bacteria such as *Prevotella* during anaerobic respiration, is related to increased intestinal inflammation (Yano et al., 2015). Cao et al. (2021) have shown a positive correlation between plasma IFN-γ level and *Pseudomonas*, *Streptomyces*, and *Clostridium* relative abundances, whereas a negative correlation between plasma IFN-γ level and *Blautia* relative abundance. Furthermore, a positive correlation between plasma IL-6 concentration and relative abundance of *Bacillus* was also observed*.* Besides, they found butyrate-producing bacteria, such as *Anaerostipes* and *Coprococcus*, were negatively correlated with IL-6 in severe ASD subjects (Cao et al., 2021). LPS, a major component of the cell wall of gram-negative bacteria, can induce the immune response of mammalian cells *via* lipid A, its toxic component. The data of Maigoro and Lee (2021) showed a significant increase in the prevalence of gram-negative bacteria in the gut of individuals with ASD compared to healthy subjects. Moreover, LPS could also be produced by gut microbiota, absorbed into the blood across an injured gut wall, and activate Toll-like receptors in the ENS and CNS (Al-Asmakh and Hedin, 2015; Garcia- Gutierrez et al., 2020). Non-canonical NLRP3 inflammasome activation by gram-negative bacteria (i.e., *Citrobacter rodentium*, *Escherichia coli*, *Legionella pneumophila*, *Salmonella typhimurium*, and *Vibrio cholerae*) further activated the transcription of IL-1β, IL-18, and NLRP3 through NF-κB activation (Pellegrini et al., 2020).

### 3.9 Bacterial Metabolites

There is growing evidence revealing that certain metabolites generated by gut microbes are responsible for ASD. Liu et al. (2021) found that the single-nucleotide variations in ASD were significantly enriched in genes correlated with the microbiome composition and a broad aspect of microbial functions, especially metabolism. Disordered compounds such as SCFAs, p-Cresol, dimethylamine, hippuric acid, and phenylacetylglutamine were detected in patients with ASD. These compounds are widely derived from gut bacteria, which induce autistic behavior by influencing and regulating the host’s immune and nervous systems. Generally, the increase of toxic metabolism and decrease of protective metabolism are observed in gut dysbiosis (Sivamaruthi et al., 2020). Several pieces of evidence have indicated that a lower abundance of protective bacteria plays critical roles in decreasing oxidative stress, cell detoxification, and excretion of heavy metal (Engwa et al., 2016; Khatua et al., 2017).

With an increasing abundance of toxin-forming bacteria in ASD children, some metabolites derived from the activity of detrimental bacteria are closely related to the ASD pathogenesis. *Clostridiaceae* is regarded to synthesize certain toxic metabolic products to humans, such as phenols, p-Cresol, and certain indole derivatives (Dawson et al., 2008). One study has confirmed that the increased *Clostridiales* is closely correlated with repetitive behaviors and GI problems in ASD, which can be rescued by using antibiotic (Finegold, 2008). In ASD patients, increased relative abundance of *Bacteroidetes* is correlative with the level of SCFAs. Macfabe (2012) have found that propionic acid or other SCFAs can lead to the biological, chemical, and pathological changes that are features of autism.

## 4 Novel Therapeutic Approaches Targeting Gut Microbiome for ASD

### 4.1 Probiotics/Prebiotics/Synbiotics

Probiotics are live microorganisms that, when ingested or applied locally in sufficient amounts, can correct the dysbiosis. *Bifidobacterium* and *Lactobacillus* strains as well as *Saccharomyces boulardii* are commonly used as probiotics. Prebiotics are non-digestible components of the human body that benefit the host health by selectively stimulating the growth and activity of gut bacteria, particularly *Lactobacillus* and *Bifidobacterium*. Prebiotics, specifically galacto-oligosaccharides (GOSs), have also shown a beneficial impact on ASD symptoms and comorbidities. Synbiotics refers to a combination of both prebiotics and probiotics (Janmohammadi et al., 2021). Rebuilding the microbiome in the gut appears to be the promising therapeutic intervention in ASD.

#### 4.1.1 Treatment With Probiotics/Prebiotics/Synbiotics in the ASD Animals

A plethora of studies on the role of probiotics in inflammation have been performed in ASD animals (Cristofori et al., 2021). One study has reported that orally given human commensal *Bacteroides fragilis* NCTC 9343 to MIA offspring effectively corrects gut permeability and microbial composition specified in diversity of *Clostridia* and *Bacteroidia* OTUs and improves ASD-like deficits (Hsiao et al., 2013). Wang et al. (2019) have further found that oral probiotics during pregnancy decreased the ASD-like behaviors induced by MIA in offspring and rescued parvalbumin positive neuron loss as well as the reduction of GABA in the prefrontal cortex of adult offspring. Consistently, one independent study also found that feeding with a probiotic *Lactobacillus* strain (*L. plantarum* L168) partially rescued the social behavior in *kdm5*-deficient flies (Chen et al., 2019). The underlying mechanism is linked to transcriptionally regulate component genes related to the immune deficiency signaling pathway, which in turn keeps host–commensal bacteria homeostasis in a demethylase-dependent manner (Chen et al., 2019). Prebiotic (propolis and bee pollen) supplements have the potential in ameliorating neuroinflammation and dysbiosis in a rodent model of autism (Aabed et al., 2019).

One recent research interest in the neurotransmitter homeostasis is a link between brain and gut microbiota. Kong et al. (2021b) assayed the effect of three *Lactobacillus* strains (*L. helveticus* CCFM1076, *L. acidophilus* La28, and *L. acidophilus* JCM 1132) on autistic-like behavioral symptoms in VPA-treated rats from weaning to sexual maturation. The authors reported that oral treatment of *L. helveticus* CCFM1076 for 4 weeks restored neurotransmitter homeostasis by improving the balance of the 5HT system in the PNS and CNS, thereby ameliorating autistic-like behaviors, while *L. acidophilus* La28 and *L. acidophilus* JCM 1132 did not (Kong et al., 2021b). One study was performed on 50 juvenile hamsters, in which the administration of a mixture of *Bifidobacteria* and *Lactobacilli strains* (ProtexinR) alleviated glutamate excitotoxicity through restoring the depleted GABA and Mg^2+^ as well as reducing glutamate (El-Ansary et al., 2018). This might explain the underlying mechanisms of probiotics involved in the improvement of autistic-like behaviors.

It is worth noting that bacterial species are sensitive to an autism-related mutation. Treatment of *L. reuteri* in *Shank3* KO mice produced beneficial effects on social and repetitive behaviors, which is correlated with altered GABA receptor levels in multiple brain regions (Tabouy et al., 2018). Consistent with this study, Sgritta1 et al. (2019) further confirmed that *L. reuteri* corrected social deficits in several tested ASD mouse models (MHFD, VPA, GF, and BTBR mice) through restoring the composition of the host’s gut microbiota, regardless of the initial insult triggering the disorder. Instead, *L. reuteri* acts in a vagus nerve-dependent manner and promotes social interaction-induced long-lasting synaptic plasticity in the mesolimbic dopamine reward system of ASD mice (Sgritta et al., 2019). In addition, one recent study confirmed that probiotic treatment could reconstitute the gut microbiome composition in Dip2a KO mice (Zhang et al., 2021).

#### 4.1.2 Treatment With Probiotics/Prebiotics/Synbiotics in the ASD Patients

Currently, several therapeutic trials have explored the efficacy of probiotics for treating ASD symptomatology (Table 3). In a randomized, double-blind, placebo-controlled trial, *Lactobacillus plantarum* PS128 (PS128) administered to boys with ASD aged 7–15 for 4 weeks ameliorated opposition/defiance behaviors (Liu et al., 2019). In line with this study, Mensi et al., 2021 found that autistic children and adolescents who received *L. plantarum* PS128 had greater improvements in terms of global functioning characterized by increased attention, communication skills, and personal autonomies. In another randomized, double-blind, placebo-controlled pilot trial, individuals with ASD aged 3–20 years who received oral probiotic *L. plantarum* PS128 and intranasal oxytocin elicited significant improvements in ASD core socio-behavioral symptoms, clinical global functioning, and gut microbiome dysbiosis (Kong et al., 2021c). It was revealed that the two treatments are supposed to have synergistic interactions in the regulation of the gut–brain axis. A case study reported by Kobliner et al. (2018) revealed that the supplementation of *S. boulardii*, a non-pathogenic probiotic yeast, successfully reduced obsessive-compulsive disorder and self-injurious behavior in a 16-year-old ASD subject.

**TABLE 3:** Microbial-based therapeutic interventions in ASD patients.

Type of trial	Treatments	Findings	References
Treatments with probiotics/prebiotics/synbiotics in ASD patients			
Case study	*S. boulardii*	Reduced obsessive compulsive disorder and self-injurious behavior	Kobliner et al. (2018)
Randomized, double-blind, placebo-controlled pilot trial	*Lactobacillus plantarum PS128 *+ intranasal oxytocin	Improved ASD core socio-behavioral symptoms, clinical global functioning, and gut microbiome dysbiosis	Kong et al. (2021c)
Randomized prospective studies	*Lactobacillus plantarum*PS128	Increased attention, communication skills, and personal autonomies	Mensi et al. (2021)
Randomized, double-blind, placebo-controlled study	*Lactobacillus plantarum*PS128	Ameliorated opposition/defiance behaviors	Liu et al. (2019)
A prospective, open-label study	*Lactobacillus acidophilus*, *Lactobacillus rhamnosus*, and *Bifidobacteria longum*	Have beneficial effects on both behavioral and GI manifestations	Shaaban et al. (2018)
Double-blind randomized, placebo-controlled trial	Probiotics (De Simone Formulation)	Improve core autism symptoms in the social-affective domain	Santocchi et al. (2020)
A randomized controlled trial	Probiotic mixture (Vivomixx®)	Alleviated autistic symptoms	Santocchi et al. (2016)
Case study	VSL#3	Improved autistic core symptoms and GI symptoms	Grossi et al. (2016)
A randomized, double-blind, placebo-controlled	B-GOS®	Improved anti-social behavior and significant increase of Lachnospiraceae family	Grimaldi et al. (2018)
A double-blind, placebo-controlled intervention study	Probiotics + FOS	Reduced the severity of autism and GI symptoms	Wang et al. (2020b)
Randomized, double-blind, crossover clinical trial	BCP + *B. infantis*	Reduced GI symptoms and aberrant behaviors	Sanctuary et al. (2019)
A placebo-controlled pilot trial	VISBIOME (eight probiotic species)	Improved parent-selected target symptoms	Arnold et al. (2019)
Multi-center clinical study	ABA training + probiotics (six strains of bacteria)	Decreased ATEC and GI scores	Niu et al. (2019)
Open-label study	Probiotic	Decreased levels of total SCFAs and lysozymes	Adams et al. (2011)
Open-label trial	*Lactobacillus acidophilus* (strain Rosell-11)	Improved the ability to concentrate and fulfil orders	Kałużna-Czaplińska and Błaszczyk, (2012)
Treatments with antibiotics in the ASD patients			
Case report	Amoxicillin	Improve speech, eye contact, and sleep behaviors and reduced repetitive behaviors	Kuhn et al. (2012)
Case reports	Vancomycin	Ameliorated communication and several behavioral defects	Sandler et al. (2020)
Treatments with FMT in the ASD patients			
Open-label clinical trial	FMT	Mitigated autism symptoms and GI disorder; reconstructed gut microbiota; and recovered the serum levels of 5-HT, GABA, and DA	Li et al. (2021)
Open-label trial	FMT	Driving the metabolic profile of the ASD group similar to the TD group	Kang et al. (2020)
Open-label study	FMT	Improved most of GI symptoms and ASD-like symptoms	Kang et al. (2017)
Follow-up study	FMT	Maintained the improved most of GI symptoms and ASD-like symptoms	Kang et al. (2019)
Open-label, randomized wait-list-controlled trial	FMT	Improved ASD-related symptoms and GI symptoms	Zhao et al. (2019)
Treatments with dietary interventions in the ASD patients			
Parallel randomized double-blind, placebo-controlled trial	Vitamin D	Alleviated CARS and ATEC scales	Javadfar et al. (2020)
Randomized clinical trial	Gluten free diet	Improved gastrointestinal symptoms and ASD behaviors	Ghalichi et al. (2016)

Likewise, one study in an Egyptian cohort of 30 autistic children indicates that 3 months of supplementation of three probiotic strains (*L. acidophilus*, *Lactobacillus rhamnosus*, and *Bifidobacteria longum*) have beneficial effects on both behavioral and GI manifestations of ASD as well as the colony counts of *Bifidobacteria* and *Lactobacilli* levels (Shaaban et al., 2018). In a double-blind, randomized, placebo-controlled trial, which was carried out on 85 preschoolers with ASD, probiotics (De Simone Formulation) containing 450 billion of eight probiotic strains was administered in 42 infants and a placebo in the remaining infants in the control group for 6 months; the outcome revealed that administration of probiotic may potentially improve core autism symptoms in the social-affective domain in a subset of ASD children independent of the specific intermediation of the probiotic effect on GI symptoms (Santocchi et al., 2020). A randomized controlled trial from Santocchi et al. (2016) has reported that individuals with ASD and GI symptoms treated with a probiotic mixture (Vivomixx®) showed dramatically alleviated autistic symptoms in behavioral profiles and in cognitive, linguistic, and adaptive functioning compared to placebo-treated individuals with ASD and GI symptoms. VSL#3, a multi-strain mixture of eight probiotics, administration in humans has been demonstrated to increase *Lactobacilli* and *Bifidobacteria* count (Rajkumar et al., 2014). One study from Grossi et al. (2016) revealed that both autistic core symptoms and GI symptoms in a boy (12 years old) with ASD and severe cognitive dysfunction were improved markedly by the supplementation of VSL#3 for 4 weeks. A pilot crossover trial has confirmed that supplementation with VISBIOME formulation containing eight probiotic species in ASD children aged 3–12 displayed significant improvement in GI complaints compared with placebo treatment (Arnold et al., 2019).

Additionally, the combined therapies address multiple aspects of the ASD. A combined dietary approach including 6-week Bimuno® galactooligosaccharide (B-GOS®) prebiotic and exclusion diet intervention in 30 autistic children resulted in a significant increase of *Lachnospiraceae* family, as well as significant changes in fecal and urine metabolites, which are related to improvements in anti-social behavior (Grimaldi et al., 2018). Similarly, one recent study was conducted to evaluate the effect of combination treatment with probiotics (a mixture of four probiotic strains) and fructo-oligosaccharide (FOS) in ASD children. It was revealed that probiotics + FOS intervention increased beneficial bacteria (*Bifidobacteriales* and *Bifidobacterium longum*) and suppressed suspected pathogenic bacteria (*Clostridium*), with marked alleviation in the severity of autism and GI symptoms (Wang et al., 2020b). Bovine colostrum product (BCP), a source of prebiotic oligosaccharides, has a beneficial effect on microbiota composition. One small pilot study was performed to explore the effect of a combination treatment (BCP + *B. infantis*) in children ages 2–11 with ASD and GI comorbidities. It has been indicated that the combination treatment is well-tolerated in this cohort and reduced GI symptoms and aberrant behaviors *via* inhibition of inflammatory factors (Sanctuary et al., 2019). One multi-center clinical study in China treated 37 children with ASD with 4 weeks of applied behavior analysis (ABA) training in combination with probiotics containing six strains of bacteria, while 28 other children with ASD were treated with ABA training alone (Niu et al., 2019). It has been confirmed that ABA training in combination with probiotics treatment brings more benefit to ASD children (Niu et al., 2019).

Metabolic modifications were also involved in the mechanism of probiotics for the treatment of ASD. One study from 58 autistic children and 39 healthy TD children found that GI symptoms were strongly correlated with the severity of autism. Probiotic oral supplementation decreased the levels of total SCFAs and lysozymes in ASD subjects (Adams et al., 2011). An open-label trial found that in 22 autistic children (age range 4–10 years) oral supplementation with *L. acidophilus* strain Rosell-11 for 2 months decreased the level of d-arabinitol, a metabolite of *Candida* species, and improved the ability to concentrate and fulfil orders (Kałużna-Czaplińska and Błaszczyk, 2012).

### 4.2 Antibiotics

Studies have implicated that antibiotics alter the gut microbiota composition (Palleja et al., 2018). Antibiotic treatment disturbs existing microbial balance by inhibiting the growth of particular components of that ecosystem. Animal studies and clinical reports showed early-life exposure to antibiotics might be involved in the ASD pathogenesis (Holingue et al., 2020). On the other hand, pieces of evidence also suggest that antibiotics have potential benefits in ameliorating ASD symptoms (Xu et al., 2019).

#### 4.2.1 Treatment With Antibiotics in ASD Animals

As mentioned above, *15q dup* mice display similar poor social communication and behavioral inflexibility to that observed in ASD patients. Neomycin treatment improved social communication in *15q dup* mice *via* increased beneficial OTUs such as *Clostridium* clusters XIVa and IV (Septyaningtrias et al., 2020). Interestingly, *kdm5*-deficient flies displayed deficits in intestinal barrier and social behavior that correlate with compositional changes in the gut microbiota. Antibiotic treatment of conventionally reared kdm5K6801/10424 flies and intestinal specific *kdm5* knockdown rescued social phenotypes and intestinal defects by modulating the gut microbiota (Chen et al., 2019). Moreover, antibiotic treatment of WT flies did not show social avoidance and social space differences in behavior (Chen et al., 2019).

#### 4.2.2 Treatment With Antibiotics in ASD Patients

A prospective birth cohort with 116 ASD cases and 860 TD child controls found that the risk for ASD was attenuated in women who experienced MIA during pregnancy and received antibiotics, suggesting that antibiotic treatment during pregnancy in the context of MIA may protect against the increased risk for ASD in the offspring (Holingue et al., 2020). There is one case report on the administration of amoxicillin to five children diagnosed with an ASD and Lyme disease for 6 months, which deemed to improve speech, eye contact, sleep behaviors, and a reduction of repetitive behaviors (Kuhn et al., 2012).

In a pivotal study, Sandler et al. (2020) treated 11 regressive-type ASD children with oral vancomycin, a useful bactericidal antibiotic for treatment of *Clostridium difficile*-associated colitis, and noticed that communication and several behavioral defects were ameliorated markedly during the 8-week treatment period. However, these effects were transient, and behavior disorder appeared after vancomycin discontinuation to treat ASD children (Sandler et al., 2020). Additionally, vancomycin-resistant enterococcus is induced by overuse, a very serious health concern that should be considered, when vancomycin is applied to treat ASD children.

### 4.3 FMT Therapy

FMT is a promising approach for manipulating the gut microbiota by transferring donor fecal suspension to patients in order to correct dysbiosis of gut microbiota in the recipients. It has been proven successful in treating recurrent *C. difficile* infection (Xu et al., 2021), inflammatory bowel disease (Bauer et al., 2020; Tan et al., 2020), irritable bowel syndrome (Myneedu et al., 2019), and several kinds of non-GI diseases (Aroniadis and Brandt, 2013). FMT can effectively adjust the gut microbial profile and restore the proportion of anti-inflammatory bacteria (Ooijevaar et al., 2019). Therefore, establishing a healthy gut microbiota using FMT is a promising new treatment for ASD (Xu et al., 2021). Importantly, the FDA has granted approval to a FMT therapy applied in ASD children in 2019.

#### 4.3.1 Mice–Mice FMT

Goo et al. (2020) found that FMT from normal mice to *Fmr1* KO mice ameliorated autistic-like behaviors, especially in cognitive impairment and defects in social novelty preference. In addition, FMT reduced the increased levels of TNFα and Iba1 in the *Fmr1* KO mouse brains and normalized *A. muciniphila* level to WT level. Likewise, one recent study has reported that FMT from WT mice improved the autistic-like behaviors in *EphB6* KO mice accompanied with the increased relative abundance of *Deferribacteres* at the phylum level and *Mucispirillum* at the genus level (Li et al., 2020). In addition, decreased vitamin B_6_ in *EphB6* KO mice is crucial for the gut microbiota-mediated autism-like behavior, which could be normalized by FMT from the WT mice (Li et al., 2020). Furthermore, abnormal social behaviors in GF mice are rescued after the mice are colonized with normal flora (Needham et al., 2018).

Studies have shown that social deficits and gut microbiota dysbiosis in MHFD offspring are inhibited by co-housing with offspring of mothers fed a regular diet (MRD) (Buffington et al., 2016). It has been reported that social behavior deficits were detected in GF mice due to lack of more bacterial species in the intestinal microbiota. FMT from MRD offspring at 4 weeks, but not at 8 weeks, rescued GF social behavior. This study indicates a critical neurodevelopmental window for microbial reconstruction and contributes to social behavior improvement (Needham et al., 2018).

#### 4.3.2 Human–Mice Inter-Species FMT

On the other hand, the effect of FMT was evaluated in one recent study using cultured gut microbiota transplantation or conventional FMT from healthy individuals to the MIA-induced ASD mouse model (Chen et al., 2020). ASD mouse model displayed significant amelioration of anxiety-like and repetitive behaviors, as well as a correction of chemokine disorders (GRO-α, MIP-1α, MCP-3, RANTES, and eotaxin) followed by transplantations with the original donor microbiota and the cultured microbiota (Chen et al., 2020). Moreover, both conventional FMT and the cultured microbiota transplantation have the ability to rescue several critical differential taxa (S24-7, Clostridiaceae, Prevotella, and Candidatus Arthromitus) in gut microbial composition of ASD mice, which is linked to serum levels of MIP-1α, MCP-3, RANTES, and eotaxin. It seemed that FMT from healthy human donors shifts the gut microbial profile in a mouse model of ASD closer to that of healthy mice (Chen et al., 2020).

#### 4.3.3 Human–Human FMT

Obviously, FMT is likely to be a valuable treatment to correct dysbiotic gut microbiota in ASD patients through transferring opportunistic pathogens or infections. The clinical trial has been investigated to explore the influence of FMT on GI and behavior symptoms in 18 patients with ASD (7–16 years old) and comorbid GI symptoms (Kang et al., 2017). It has been revealed that 8-week FMT treatment improved most of the GI symptoms such as constipation, diarrhea, indigestion, and abdominal pain as well as ASD-related symptoms in 16 out of 18 ASD children (Kang et al., 2017). Different from vancomycin therapy, benefits produced by FMT were sustained at least 8 weeks after the treatment. Coincident with these clinical improvements, bacterial and phage deep sequencing analyses revealed that FMT increased the overall bacterial diversity and the potentially beneficial microbe abundance in the recipients, and these changes persisted for 8 weeks (Kang et al., 2017). These data indicate that FMT successfully shifts the gut microbiota of children with ASD toward that of healthy controls and their donors. This study sheds light on the potential of targeting gut microbiome for ASD treatment *via* restoring a healthy microbiota composition.

Moreover, a follow-up study at 2 years post-treatment was conducted and found that GI symptom improvements in most participants were maintained, and the autism symptoms had continued to improve since the end of treatment (Kang et al., 2019). Importantly, the gut microbial community diversity and the relative commensal bacterial abundances of two bacterial genera, *Bifidobacteria* and *Prevotella*, in ASD children were significantly increased after FMT treatment and remained pretty similar to those in TD children at 2 years after treatment (Kang et al., 2019). Besides, an open-label, randomized, wait-list-controlled trial performed by Zhao et al. (2019) showed that FMT treatment temporarily improved ASD-related symptoms and GI symptoms 2 months following two FMTs in 24 ASD children compared to 24 control ASD children. Additionally, FMT therapy typically reduced the abundance of *Bacteroides fragilis* and persistently shaped the gut microbiota profile of ASD individuals to a healthy state.

Meanwhile, it has been reported that FMT treatment shifted plasma metabolite profiles in the ASD children to resemble more closely those of their TD peers (Kang et al., 2020). Likewise, in a recent study within a cohort of 18 ASD with GI symptoms and 20 TD children with no history of GI symptoms, FMT treatment induced global alterations in plasma profiles across diversified metabolic traits, such as nicotinate/nicotinamide and purine metabolism (Qureshi et al., 2020). In addition, FMT treatment caused driving the metabolic profile of the ASD group similar to the TD group. For 669 fecal metabolites detected, FMT treatment decreased *p*-Cresol sulfate levels in children with ASD similar to those in TD children (Qureshi et al., 2020). Due to striking heterogeneity in stool, the effect of FMT treatment on fecal metabolites will be re-valuated with a larger patient cohort and a placebo arm. Importantly, in a recent clinical trial involving 40 ASD (age 3–17 years) with GI symptoms and 16 TD children with no history of GI symptoms, FMT treatment mitigated autism symptoms and GI disorder, reconstructed gut microbiota, as well as recovered the serum levels of several neurotransmitters such as 5-HT, GABA, and DA in the ASD cohort (Li et al., 2021).

There are some challenges needed to be considered when FMT treatments were used to treat ASD. Dosage, duration of treatment, as well as use of antibiotics and bowel-cleansing regimes before treatment need to be determined with a larger population size and standardized clinical trial (Zhang et al., 2020). The first thing to be considered is that donors need to be screened before donation to minimize the risk of transferring opportunistic pathogens or infections to recipients.

### 4.4 Dietary Interventions

There is a growing body of scientific evidence that environmental factors such as diet affect GI microbiota composition. Therefore, dietary interventions may prove an easy approach to alter gut microbiota for neuropsychiatric patient treatment. Clinical trials have found that dietary patterns impact fecal microbiota composition and dynamic change in children with ASD (Berding and Donovan, 2018, 2020). Instead, one recent study indicated that ASD-related restricted behaviors might cause dietary restrictedness and in turn lead to decreased taxonomic diversity and looser stool consistency (Yap et al., 2021). The correlations between diet and fecal microbiota composition need to be further explored.

Vitamin D is essential for CNS development. Findings from previous studies have strengthened the link between vitamin D deficiency and the risk of autism. It has been reported that propionic acid occurs naturally in some foods and acts as a metabolic product of gut microbiota involved in the development of ASD. Alfawaz et al. (2014) further found that vitamin D displayed a greater protective than therapeutic effect on brain intoxication caused by propionic acid in rats. In line with this, a clinical study has indicated that vitamin D supplementation may alleviate symptoms of ASD when 25(OH)D levels in the serum were increased markedly (Javadfar et al., 2020). Polyunsaturated fatty acids (PUFAs) are important constituents of phospholipids. N-3 PUFA (docosahexaenoic acid) and n-6 PUFA (arachidonic acid) formed brain cellular membranes, are provided by dietary supply, and are critical for brain development evidenced by aggregation in embryonic and post-natal brains. Of note, evidence has shown that the state of n-3 and n-6 PUFAs also influences the gut microbiota, thereby improving autism-like behaviors. Wang et al. (2020a) also found the n-3/n-6 (1:5) diet improved fecal microbiota composition in VPA-exposed rats characterized by the increased microbial abundance and reduced *Firmicutes*. It is supposed that appropriate n-3/n-6 PUFA ratio intake is a promising intervention for treating ASD. Another study has also confirmed that orally supplemented omega-3 fatty acids and vitamin B12 combination were more efficacious in treating autism by improvements of oxidative stress and abnormal lipid metabolic as well as remodeling microbial communities (Alfawaz et al., 2018).

Furthermore, exclusion approaches, such as gluten- and casein-free diets (GFCF), had a favorable impact on ASD-related symptoms. In one randomized clinical trial, intaking of GF diet improved GI symptom and ASD-related behavioral disorders (Ghalichi et al., 2016). A number of studies have addressed the beneficial effects of ketogenic diet composed of low carbohydrate, adequate protein, and high fat on improvement of autism-like behaviors *via* reshaping microbial composition (Newell et al., 2016; Rawat et al., 2021). There is still a lack of clinical data to support the correlation of observed behavioral improvements and GI symptoms produced by GFCF and ketogenic diet.

There are currently no randomized placebo-controlled clinical trials using dietary intervention, and this might be interpreted by the truth that individuals diagnosed with neuropsychiatric conditions intake different types of medications interfering with microbial communities. Although there are promising preliminary studies, further confirmative studies should be conducted to examine the extent by which past food habits of ASD patients and how the dietary interventions influence the pathogenesis and therapy of ASD.

## 5 Conclusion and Challenges

Despite discrepancies between studies, the close interaction of the gut microbiota with the physical condition of ASD patients indicates that abnormal microbiota composition may aggravate the behavioral symptoms and biological signs of ASD. Differences in microbiota composition remain uncertain due to differences in methodology, study population, and confounding factors. The manipulation of the gut microbiome seems to be a promising therapy to mitigate ASD-associated core symptoms and behavioral abnormalities in ASD subjects. Future studies should systematically investigate the microbial composition of children with ASD and stress the importance of interpreting a close association between typical bacterial species and ASD symptoms. Treatment using targeted bacterial strain is important, as the effects of probiotic bacteria can be highly strain specific. In addition, new therapeutic measures will be of key focus on providing an early intervention strategy to decrease the severity of the disease in ASD children. Longer follow-up of clinical course would help to further determine its efficacy and safety.
